# Maternal copper levels moderate the neuroprotective effect of zinc on early childhood development: evidence from a birth cohort and proteomic profiling

**DOI:** 10.3389/fnut.2026.1868249

**Published:** 2026-07-13

**Authors:** Chaoqun Liu, Zhuoqing Zhang, Mingtian Huang, Hong Cheng, Caitong He, Likai Zhou, Yu Zhang, Jijie Zhang, Sisi Wei, Tao Liang, Xiaobo Yang

**Affiliations:** 1 Center for Genomic and Personalized Medicine, Guangxi key Laboratory for Genomic and Personalized Medicine, Guangxi Collaborative Innovation Center for Genomic and Personalized Medicine, Guangxi Medical University, Nanning, Guangxi, China; 2 School of Public Health, Guangxi Medical University, Nanning, Guangxi, China; 3 Department of Obstetrics, Maternal & Child Health Hospital of Nanning, Nanning, Guangxi, China; 4 Department of Science and Education, Maternal & Child Health Hospital of Yulin, Yulin, Guangxi, China; 5 Department of Health Care, Maternal & Child Health Hospital of Liuzhou, Liuzhou, Guangxi, China; 6 Department of Science and Education, Maternal & Child Health Hospital of Qinzhou, Qinzhou, Guangxi, China; 7 Department of Medical Services Section, Maternal & Child Health Hospital of Guigang, Guigang, Guangxi, China; 8 Department of Pediatrics, Maternal & Child Health Hospital of Wuzhou, Wuzhou, Guangxi, China; 9 Department of Occupational Health and Environmental Health, School of Public Health, Guangxi Medical University, Nanning, Guangxi, China

**Keywords:** neurodevelopment, zinc, copper, copper/zinc ratio, birth cohort, proteomics

## Abstract

**Background & aims:**

The impact of maternal zinc-copper (Zn-Cu) homeostasis on offspring brain development remains underexplored. This study investigated the relationships between maternal zinc (Zn), copper (Cu), and the Cu/Zn ratio during mid-pregnancy and subsequent early childhood neurodevelopment, utilizing both a prospective birth cohort and an *in vivo* rodent model to elucidate the underlying biological mechanisms.

**Methods:**

This prospective cohort study recruited 725 mother-child pairs from the Guangxi Birth Cohort Study (GBCS). Multivariable linear regression models, restricted cubic splines (RCS), and formal interaction tests with stratified analyses were employed to evaluate the non-linear dose-response relationships and metal-metal interactions influencing the child's developmental quotient (DQ). To explore the underlying mechanisms and provide biological plausibility for the epidemiological findings, pregnant rats were divided into four dietary groups with varying Cu/Zn ratios. Offspring spatial cognitive behaviors were assessed via the Morris Water Maze (MWM), followed by high-throughput hippocampal proteomics.

**Results:**

Maternal Cu and the Cu/Zn ratio exhibited significant inverse non-linear associations with child neurodevelopment across multiple domains. Crucially, the neuroprotective potential of Zn was significantly moderated by maternal Cu status; Zn was positively associated with developmental scores under low Cu conditions, but this benefit was progressively attenuated at higher Cu levels. In the MWM test, despite comparable baseline spatial learning boundaries, the Medium Cu/Zn ratio group displayed significantly altered exploratory patterns during the spatial probe trials. Proteomic analysis revealed a universal downregulation of the glutamate transporter SLC1A3 and significant dysregulation of critical synaptic markers (including SYT1, KCC2, and MAP2) across all Cu/Zn imbalance groups.

**Conclusions:**

The maternal mid-pregnancy Cu/Zn ratio is non-linearly associated with offspring neurodevelopment, potentially mediated by hippocampal synaptic protein alterations. These findings provide a preliminary conceptual framework advocating for personalized prenatal nutritional assessments, which should integrate dual-index monitoring and consider regional geochemical baselines to optimize intervention strategies.

## 1 Introduction

Maternal nutritional integrity is a primary determinant of progeny development, particularly during the foundational first 1,000 days (from pregnancy to 2 years of age) (1–3). The fetal stage is a highly sensitive period characterized by intensive neuronal generation, migration, connectivity, and differentiation (4). As essential trace elements, zinc (Zn) and copper (Cu) play indispensable roles in fetal programming and infant neurodevelopment (4–6). However, elevated concentrations of these metals are associated with neurotoxicity (7). Given this duality, the intricate interplay and homeostasis between zinc and copper rather than their absolute concentrations alone remain poorly understood. Elucidating how this balance modulates neural development and identifying its operative mechanisms is therefore essential.

Zn is an essential trace element and cofactor for over 300 enzymes (8). It serves as a critical regulator of central nervous system development, influencing brain morphogenesis, stem cell dynamics, neuronal number, and cerebellar myelination (9, 10). Furthermore, it modulates molecular processes including gene expression, N-methyl-D-aspartate (*NMDA*) receptor expression, and the organization of synaptic scaffold proteins (11–14). Cu is an essential cofactor for energy and redox metabolism enzymes and a key regulator of neurotransmission via α-amino-3-hydroxy-5-methyl-4-isoxazolepropionic acid (*AMPA*) and NMDA receptors (15, 16). During neural development, the brain's metabolic demands increase significantly, and energy conversion is highly active, rendering the developing brain extremely susceptible to copper deficiency or excess (17, 18). Excessive intracellular copper can also interfere with zinc protein metalation. Cu/Zn superoxide dismutase (Cu/Zn SOD) plays a vital role in antioxidant defense. Its proper function relies on the copper-zinc ratio (Cu/Zn ratio), rather than the absolute levels of the individual metals, to maintain catalytic efficiency. Current studies link the Cu/Zn ratio to multiple diseases, highlighting its potential role as a biomarker of systemic dysfunction (19–22). Although the neuroprotective potential of prenatal zinc is widely recognized, evaluating its effects in isolation may overlook complex nutrient-nutrient interactions. In our team's previous prospective cohort study exploring multi-metal exposures (23), zinc was not optimally integrated into the initial developmental models due to analytical detection limits tailored for heavy metals. Upon optimizing our whole-blood analytical framework in the current study, preliminary single-element analysis of maternal zinc revealed inconsistent and non-linear associations with child developmental scores. This prompted us to shift our focus toward metal-metal interactions. Biochemically, copper and zinc form a classic physiological antagonistic pair; they share common intestinal transporters and mutually regulate metallothionein synthesis. Evaluating zinc alone may introduce bias by ignoring the baseline status of its natural antagonist. Indeed, recent epidemiological evidence, such as findings from the Italian PHIME cohort (7), emphasizes the critical importance of evaluating the joint status and balance of copper and zinc in cord blood and maternal profiles for early neurodevelopment.

However, epidemiological evidence regarding the impact of the maternal Cu/Zn ratio during pregnancy on neurodevelopmental outcomes in offspring remains limited and inconsistent. Therefore, in the current study, the relationships between maternal Zn, Cu, and the Cu/Zn ratio during mid-pregnancy and subsequent child neurodevelopment were comprehensively analyzed. Concurrently, *in vivo* animal experiments were conducted to elucidate the underlying biological mechanisms.

## 2 Materials and methods

### 2.1 Population-based prospective cohort study

#### 2.1.1 Study design and participants

This study was embedded within the Guangxi Birth Cohort Study (GBCS), an ongoing multicenter prospective cohort designed to examine prenatal environmental exposures and subsequent child health outcomes (23, 24). Pregnant women were recruited from eight maternal and child health hospitals across Guangxi, China. Notably, the Guangxi region is characterized by extensive karst topography and abundant non-ferrous metal resources, which contribute to a naturally high geochemical background of trace elements in the local environment and population. A total of 5,541 mother–infant pairs completed the first follow-up. During the second follow-up period from July to September 2018, a simple random sampling strategy was employed to recruit participants from the original cohort. A total of 1,076 children aged 2–3 years were successfully followed up. Among them, 804 children completed the neurodevelopmental assessment using the Gesell Developmental Schedules.

Eligibility criteria for the original cohort included maternal age ≥18 years, residence in the study area for more than two years, and no history of severe cardiac, hepatic, renal, or psychiatric disorders. For the current analysis, mother–child pairs were required to have complete data on maternal whole blood trace element concentrations and child neurodevelopmental assessments. Participants were excluded if they lacked essential biological samples or neurodevelopmental assessments. Furthermore, participants with extreme trace element values (>3 SD from the mean) were excluded to prevent undue leveraging effects on the restricted cubic spline (RCS) curve fitting, thereby ensuring the models accurately reflected the general population's exposure spectrum. After strictly applying these inclusion and exclusion criteria, 725 mother–child pairs were included in the final analysis. Ethical approval was obtained from the Ethics Committee of Guangxi Medical University, and written informed consent was provided by all participants. The participant selection process is detailed in Supplementary Figure S1.

#### 2.1.2 Neurodevelopmental assessment

Child neurodevelopment was evaluated using the Gesell Developmental Schedules (GDS), a comprehensive and widely recognized psychomotor assessment tool that has been extensively validated and standardized for the Chinese pediatric population. All assessments were conducted in designated quiet examination rooms at the maternal and child health hospitals by trained and certified pediatric developmental specialists. To minimize observational bias, the examiners were strictly blinded to the maternal trace element profiles. Each assessment session lasted approximately 30–45 min, adhering strictly to standardized clinical protocols and quality-control procedures.

The GDS evaluates child neurocognitive and motor trajectories across five independent functional domains: gross motor, fine motor, adaptive behavior, language, and personal-social behavior. For each domain, a Developmental Quotient (DQ) is calculated utilizing the formula: DQ = (Developmental Age / Chronological Age) × 100. In accordance with established clinical diagnostic criteria, a DQ score of < 85 in any respective domain was categorized as suboptimal development (developmental delay), whereas a score of ≥ 85 indicated normal neurodevelopmental status.

#### 2.1.3 Laboratory analysis and quality control

Maternal venous blood samples (10 ml) were collected during routine prenatal examinations at a mean gestational age of 19.0 ± 6.6 weeks. According to the standard biobanking protocol of the cohort, multiple blood fractions were prepared on-site: an aliquot of uncoagulated whole blood was specifically preserved in trace-metal-free tubes for direct trace element analysis, while the remaining volume was processed and centrifuged to separate serum, plasma, and blood cells for other cohort assessments. In the current study, whole blood zinc and copper concentrations were exclusively used and measured via inductively coupled plasma mass spectrometry (ICP-MS; iCAP Q, Thermo Scientific, Germany). All samples were analyzed by a single technician.

We validated measurement accuracy using stringent quality control protocols throughout the analysis. The specific LODs for Cu and Zn were 0.0028 μg/L and 0.0253 μg/L, respectively. Concentrations below the LOD were imputed as LOD/ √2 (25). Calibration curves were constructed using six standards (0.55–110 μg/L), and only curves with *R*^2^ > 0.999 were accepted. Analytical accuracy and precision were assessed using certified reference materials (ClinChek^®^ Serum Controls Levels 1 and 2; SRM 2670A; SRM 1640A). Inter-assay and intra-assay variability were monitored throughout the analytical process.

### 2.2 Animal experiment

#### 2.2.1 Animals and ethics statement

Specific pathogen-free Sprague–Dawley rats were obtained from the Animal Experiment Center of Guangxi Medical University. Animals were housed under controlled temperature (22 ± 2 °C), humidity (55 ± 5%), and a 12 h light/dark cycle, with free access to deionized water and food. All procedures were approved by the Animal Ethics Committee of Guangxi Medical University and complied with national guidelines for laboratory animal care (26).

#### 2.2.2 Experimental design and dietary intervention

After baseline acclimatization and successful mating, the pregnant dams were randomly assigned to one of four dietary groups (*n* = 12 per group) and maintained on experimental diets throughout gestation and lactation. Diets were based on the AIN-93G formulation, with precise modifications made exclusively to the zinc (Zn) and copper (Cu) concentrations to manipulate the maternal systemic balance (27).

The dietary doses were selected based on established nutritional standards and previous experimental evidence to represent physiologically relevant Cu/Zn imbalance conditions. (1) Control Group (Zn 30 mg/kg, Cu 6 mg/kg, theoretical Cu/Zn ratio 1:5): dosages were set strictly according to the NRC and AIN-93G standards to represent the optimal homeostatic ratio (~5:1) for rodent development (27, 28). (2) Low Cu/Zn ratio Group (Zn 30 mg/kg, Cu 0.3 mg/kg, theoretical Cu/Zn ratio 1:100): this group was designed to model severe dietary copper deficiency The Cu level (0.3 mg/kg) was far below the minimum requirement, sufficient to inhibit Cu-dependent enzyme activity without inducing immediate mortality (29). (3) Medium Cu/Zn ratio Group (Zn 100 mg/kg, Cu 6 mg/kg, theoretical Cu/Zn ratio 1:16.7): this formulation represented a Zn-induced secondary copper deficiency model. While dietary Cu remained adequate, we elevated Zn levels to 3.3 times the requirement. This design upregulates intestinal metallothionein (MT) to trap Cu and block absorption, effectively mimicking the antagonism seen in excessive Zn supplementation (30, 31). (4) High Cu/Zn ratio Group (Zn 30 mg/kg, Cu 30 mg/kg, theoretical Cu/Zn ratio 1:1): This group simulated a chronic copper overload scenario. The Cu dosage (5-fold of requirement) disrupted the physiological Zn/Cu molar ratio (shifting to 1:1), to mimic oxidative stress conditions relevant to environmental exposure (32).

Offspring were weaned at postnatal day 21 (PND21), and male pups were selected for subsequent behavioral and proteomic analyses to avoid the interference of the estrous cycle.

#### 2.2.3 Morris water maze (MWM) test

Spatial learning and memory were assessed using the Morris Water Maze on postnatal days 28–33 (*n* = 6 per group) (33). The apparatus consisted of a circular pool (1.5 m diameter) filled with water (25 ± 1 °C) made opaque with non-toxic black ink. A hidden platform was submerged 1.5 cm below the water surface in the target quadrant. The test included two phases: (1) Orientation Navigation Trial (Days 1–5): Rats were trained for 5 consecutive days with 4 trials per day. The time taken to locate the platform (escape latency) was recorded. If a rat failed to find the platform within 60 s, it was guided to it and allowed to stay for 10 s. (20 Spatial Probe Trial (Day 6): The platform was removed, and rats were allowed to swim freely for 60 s. The number of platform crossings and the time spent in the target quadrant were recorded to evaluate memory retention. Trajectories were analyzed using a video tracking system (Smart 3.0, Panlab, Spain). For the Morris water maze hidden platform training, escape latency and swimming speed across the 5 consecutive days were analyzed using a two-way Repeated Measures ANOVA (RM-ANOVA) to evaluate the main effects of time, dietary group, and their interaction. Data from the spatial probe test (platform crossings and time in the target quadrant) were analyzed using one-way ANOVA.

#### 2.2.4 TMT-based quantitative proteomics

Whole brain tissues were collected from offspring (*n* = 3 biological replicates per group), and total protein was extracted using lysis buffer containing protease inhibitors. Protein concentration was determined by the BCA method. (1) Digestion and Labeling: protein samples (100 μg) were digested with trypsin (Promega) using the Filter Aided Sample Preparation (FASP) method (34). The resulting peptides were labeled using a TMT10plex™ Isobaric Label Reagent Set (Thermo Fisher Scientific) according to the manufacturer's instructions. (2) LC-MS/MS Analysis: the labeled peptides were fractionated and analyzed on a Q Exactive™ HF-X mass spectrometer (Thermo Fisher Scientific) coupled with an Easy-nLC 1,200 system. Data were acquired in a data-dependent acquisition (DDA) mode. (3) Bioinformatics: raw data were searched against the Rattus norvegicus UniProt database using Proteome Discoverer 2.4. Differentially expressed proteins (DEPs) were identified based on a threshold of *P* < 0.05 and fold change > 1.3 (or < 0.77). Functional enrichment analyses (GO and KEGG) were performed to annotate the biological functions of the DEPs.

### 2.3 Hippocampal proteomic analysis

#### 2.3.1 Protein extraction and digestion

Hippocampal tissues from offspring rats (*n* = 3 biological replicates per group) were homogenized in lysis buffer, and protein concentrations were determined using the BCA assay. Following trypsin digestion via the filter-aided sample preparation (FASP) protocol, the resulting peptides were desalted and quantified for mass spectrometry analysis.

#### 2.3.2 DIA orbitrap astral

Peptides were analyzed using a Data-Independent Acquisition (DIA) strategy on an Orbitrap Astral mass spectrometer (Thermo Fisher Scientific). This next-generation instrument ensures high-throughput and high-sensitivity protein quantification. Peptides were separated on a reversed-phase analytical column with a gradient of increasing acetonitrile. The Astral analyzer operated in DIA mode, covering a wide precursor m/z range with optimized window widths to achieve extensive proteome coverage.

#### 2.3.3 Bioinformation

The raw DIA data acquired from the Orbitrap Astral platform were processed using Spectronaut (version 18.0 or equivalent) for automated peptide identification and quantification. Data were searched against the Rattus norvegicus UniProt database, with a false discovery rate (FDR) threshold set at 1% at both the precursor and protein levels to ensure high-confidence identifications.

Functional characterization of differentially expressed proteins (DEPs) was conducted through multidimensional bioinformatic analyses. Gene Ontology (GO) annotation was performed to classify DEPs into three categories: biological processes, molecular functions, and cellular components. The Kyoto Encyclopedia of Genes and Genomes (KEGG) database was used to identify significantly enriched metabolic or signaling pathways. To identify the “common molecular signature” of Zn/Cu imbalance across different exposure levels, an UpSet plot was used to visualize the intersection of DEPs from the Low, Medium, and High Cu/Zn ratio groups. Protein-protein interaction (PPI) networks and dot plots were generated using R-based packages (e.g., ggplot2, clusterProfiler) to investigate the functional clusters associated with neurodevelopmental outcomes.

### 2.4 Statistical analysis

#### 2.4.1 Population data analysis

Continuous variables were expressed as mean ± standard deviation (SD) or median with interquartile range (IQR), while categorical variables were presented as frequencies and percentages. Multivariable linear regression models were used to estimate the associations between maternal trace elements (Zn, Cu, and Zn/Cu ratio) and child DQ scores. Model 1 was adjusted for child age and sex; Model 2 further incorporated maternal age, pre-pregnancy BMI, educational level, household income, passive smoking during pregnancy, and child birth head circumference.

To minimize potential bias from missing data in key covariates (e.g., maternal BMI, household income), multiple imputation (MI) was performed under the missing at random (MAR) assumption. Five imputed datasets were generated using the MICE package in R (35), and the pooled estimates were utilized for the final models. Generalized linear models (GLM) and restricted cubic splines (RCS) were used to evaluate associations. To characterize potential non-linear dose-response relationships, RCS were implemented with four knots positioned at the 5th, 35th, 65th, and 95th percentiles (36). To formally assess these trajectories, exact *P*-values for overall association (*P*_overall_) and non-linearity (*P*_non−linear_) were calculated. Furthermore, to quantify the potential regulatory or modifying effects between trace elements, a multiplicative interaction term (whole-blood zinc × whole-blood copper) was integrated into the fully adjusted regression models. Stratified analyses were subsequently conducted based on the baseline maternal copper strata to evaluate specific risk patterns. Statistical significance was defined as a two-sided *P* < 0.05.

#### 2.4.2 Analysis of quantitative proteomics data

For the DIA-based proteomics data acquired via the Orbitrap Astral system, protein intensities were log_2_-transformed and normalized using the median centering method to ensure intra-group and inter-group comparability. Differentially expressed proteins (DEPs) between the experimental groups (low, medium, and high Zn/Cu ratio group) and the Control group were identified using moderated *t*-tests within the limma package. The significance thresholds were strictly set at a two-sided *P* < 0.05 and an absolute fold change |log_2_FC| > 0.38. All raw data processing and statistical filtering were performed using R software (version 4.5.1).

#### 2.4.3 Bioinformatic intersection

To investigate the biological significance of the identified DEPs, functional annotation and pathway enrichment were conducted. Gene Ontology (GO) analysis was used to categorize proteins into biological processes (BP), molecular functions (MF), and cellular components (CC). Kyoto Encyclopedia of Genes and Genomes (KEGG) pathway enrichment was used to identify activated or inhibited signaling cascades.

To pinpoint the most robust molecular markers, an UpSet plot was generated to identify the intersection of DEPs across all three exposure levels, defining the “core proteomic signature.” Protein-Protein Interaction (PPI) networks were constructed using the STRING database (version 12.0) with a confidence score > 0.4 and visualized via Cytoscape to identify key hub proteins. Enrichment was considered significant if the Benjamini–Hochberg adjusted *P*-value was less than 0.05.

## 3 Result

### 3.1 Association of maternal Cu/Zn ratio with child neurodevelopment

#### 3.1.1 Descriptive characteristics of the study population

The general characteristics of the 725 mother-child pairs included in the final analysis are presented in Table 1. The mean maternal age was 28.6 ± 4.6 years, and the average gestational age at sampling was 19.2 ± 6.7 weeks. The majority of participants had a college education or above (42.1%). To further explore potential confounders, demographic characteristics were stratified by maternal whole blood copper tertiles (Supplementary Table S2). The distributions of maternal whole blood copper and zinc concentrations are presented in Supplementary Table S3. Both metals were detected in 100% of samples, with median concentrations of 1418.19 μg/L for copper and 5123.99 μg/L for zinc, resulting in a median Cu/Zn ratio of 0.26.

**Table 1:** General characteristics of the study participants (*n* = 725).

Variable	Total (*n* = 725)
Maternal characteristics	
Age (years)	28.6 ± 4.6
Pre-pregnancy BMI (kg/m^2^)	20.6 ± 3.1
Educational level, *n* (%)	
Junior high school or below	209 (28.8)
Senior high school	211 (29.1)
College or above	305 (42.1)
Annual household income, *n* (%)	
Low	423 (58.3)
Medium	241 (33.2)
High	61 (8.4)
Alcohol use before pregnancy, *n* (%)	
Yes	193 (26.6)
No	532 (73.4)
Passive smoking during pregnancy, *n* (%)	
Yes	92 (12.7)
No	633 (87.3)
Gestational week at sampling	19.2 ± 6.7
Child characteristics	
Sex (male), *n* (%)	442 (61.0)
Gestational age at delivery (weeks)	38.6 ± 1.4
Age (years)	2.6 ± 0.1
Birth head circumference (cm)	32.8 ± 1.6
Developmental quotient (DQ)	
Adaptive behavior	86.9 ± 10.9
Gross motor	93.0 ± 12.0
Fine motor	96.6 ± 15.3
Language	84.5 ± 15.6
Personal-social behavior	94.6 ± 16.4
Total DQ	91.1 ± 11.2

Values are presented as mean ± SD for continuous variables and n (%) for categorical variables.

#### 3.1.2 Associations between maternal Cu/Zn ratio and developmental outcomes

**Figure 1:**
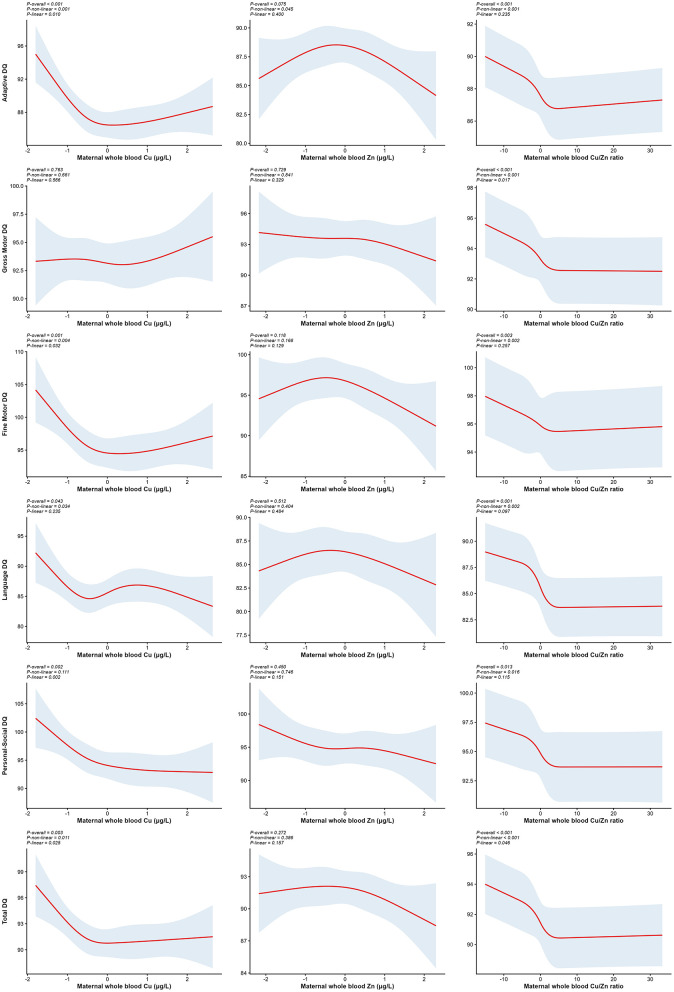
Dose-response associations of whole blood copper, zinc, and Cu/Zn ratio with neurodevelopmental quotients (DQs). The associations were evaluated using restricted cubic spline (RCS) regression models with four knots placed at the 5th, 35th, 65th, and 95th percentiles. The solid red lines represent the adjusted predicted DQ scores, and the blue shaded areas indicate the 95% confidence intervals (CIs). Models were adjusted for child age, sex, maternal education level, and gestational age. P-values for overall association (P_overall_), non-linearity (P_non−linear_), and linearity (P_linear_) are presented within each panel. Cu, copper; Zn, zinc; DQ, developmental quotient; CI, confidence interval.

Restricted cubic spline (RCS) analysis (Figure 1) revealed significant non-linear relationships between the maternal whole blood Cu/Zn ratio and all child neurodevelopmental domains (all *P*_*overall*_ < 0.05). The Cu/Zn ratio exhibited a significant inverse non-linear association, where higher ratios correlated with a steep decline in in the Adaptive, Fine Motor, and Total DQ domains (all *P*_*non*−*linear*_ < 0.01). Independent maternal Cu levels also demonstrated pronounced non-linear negative associations with Adaptive (*P*_non−linear_ < 0.001) and Fine Motor scores (*P*_non−linear_ = 0.004). Evaluation of the RCS curves (*P*_*overall*_ < 0.05, P_non−linear_ < 0.01) within the actual distribution of our data indicated a continuous, monotonically decreasing trend rather than a distinctly bounded U-shaped threshold. This pattern suggests that across the higher-exposure spectrum observed in this region, elevated Cu/Zn ratios are consistently associated with progressive declines in neurodevelopmental scores.

Multivariable logistic regression analysis (Table 2). further confirmed that higher maternal Cu levels during mid-pregnancy significantly escalated the risk of suboptimal developmental outcomes (DQ < 85). Compared with the lowest tertile (T1), children in the highest maternal Cu tertile (T3) faced an increased risk of suboptimal adaptive development (OR = 1.69, 95% CI: 1.16–2.48). For Fine Motor development, both the middle and highest tertiles were associated with increased risks (T2: OR = 1.96, 95% CI: 1.23–3.16; T3: OR = 1.70, 95% CI: 1.06–2.76). In contrast, higher Cu levels were associated with a lower risk of suboptimal Gross Motor scores (T3: OR = 0.63, 95% CI: 0.41–0.96).

**Table 2:** Adjusted odds ratios and 95% confidence intervals for the associations of whole blood zinc, copper, and Cu/Zn ratio tertiles with the risk of suboptimal developmental scores.

Exposure	Outcome	T1 (Ref)_OR (95% CI)	T1 (Ref)_*P*_value	T2_OR (95% CI)	T2_ P_value	T3_OR (95% CI)	T3_ *P*_value
Whole blood Zn	Adaptive	1.00 (Ref)	–	1.08 (0.73, 1.60)	0.691	1.27 (0.86, 1.87)	0.232
Whole blood Zn	Gross motor	1.00 (Ref)	–	1.00 (0.64, 1.57)	0.984	1.39 (0.90, 2.14)	0.138
Whole blood Zn	Fine motor	1.00 (Ref)	–	1.05 (0.66, 1.68)	0.829	1.11 (0.69, 1.78)	0.666
Whole blood Zn	Language	1.00 (Ref)	–	0.83 (0.57, 1.21)	0.335	0.92 (0.63, 1.34)	0.654
Whole blood Zn	Personal-social	1.00 (Ref)	–	1.02 (0.65, 1.61)	0.924	1.47 (0.95, 2.28)	0.086
Whole blood Zn	Total DQ	1.00 (Ref)	–	0.92 (0.60, 1.41)	0.7	1.10 (0.73, 1.67)	0.651
Whole blood Cu	Adaptive	1.00 (Ref)	–	1.44 (0.99, 2.12)	0.06	1.66 (1.14, 2.45)	0.009^*^
Whole blood Cu	Gross motor	1.00 (Ref)	–	0.79 (0.52, 1.19)	0.264	0.61 (0.39, 0.93)	0.022^*^
Whole blood Cu	Fine motor	1.00 (Ref)	–	1.92 (1.20, 3.11)	0.007^*^	1.67 (1.04, 2.73)	0.037^*^
Whole blood Cu	Language	1.00 (Ref)	–	1.15 (0.80, 1.67)	0.442	1.23 (0.85, 1.78)	0.274
Whole blood Cu	Personal-social	1.00 (Ref)	–	1.09 (0.71, 1.67)	0.692	1.09 (0.71, 1.68)	0.698
Whole blood Cu	Total DQ	1.00 (Ref)	–	1.11 (0.74, 1.68)	0.607	1.12 (0.74, 1.69)	0.601
Cu_Zn_ratio	Adaptive	1.00 (Ref)	–	1.50 (1.02, 2.20)	0.038^*^	1.11 (0.76, 1.63)	0.595
Cu_Zn_ratio	Gross motor	1.00 (Ref)	–	0.98 (0.65, 1.49)	0.933	0.74 (0.48, 1.13)	0.168
Cu_Zn_ratio	Fine motor	1.00 (Ref)	–	1.18 (0.76, 1.85)	0.46	0.77 (0.48, 1.23)	0.268
Cu_Zn_ratio	Language	1.00 (Ref)	–	1.24 (0.86, 1.80)	0.253	1.08 (0.75, 1.57)	0.674
Cu_Zn_ratio	Personal-social	1.00 (Ref)	–	1.41 (0.91, 2.19)	0.123	1.30 (0.84, 2.03)	0.233
Cu_Zn_ratio	Total DQ	1.00 (Ref)	–	1.25 (0.83, 1.90)	0.286	1.10 (0.72, 1.66)	0.662

Suboptimal developmental outcome was defined as a developmental quotient (DQ) score < 85. The lowest tertile (T1) of each whole blood metal parameter was set as the reference group. The whole blood Cu/Zn ratio was calculated by dividing the whole blood copper concentration by the whole blood zinc concentration. Multivariable logistic regression models were fully adjusted for child age, sex, maternal education, gestational age, maternal body mass index (BMI), household income, and maternal age. CI, confidence interval; Cu, copper; DQ, developmental quotient; OR, odds ratio; Ref, reference; T, tertile; Zn, zinc. Statistically significant P-values (< 0.05) are indicated with an asterisk (^*^).

**Figure 2:**
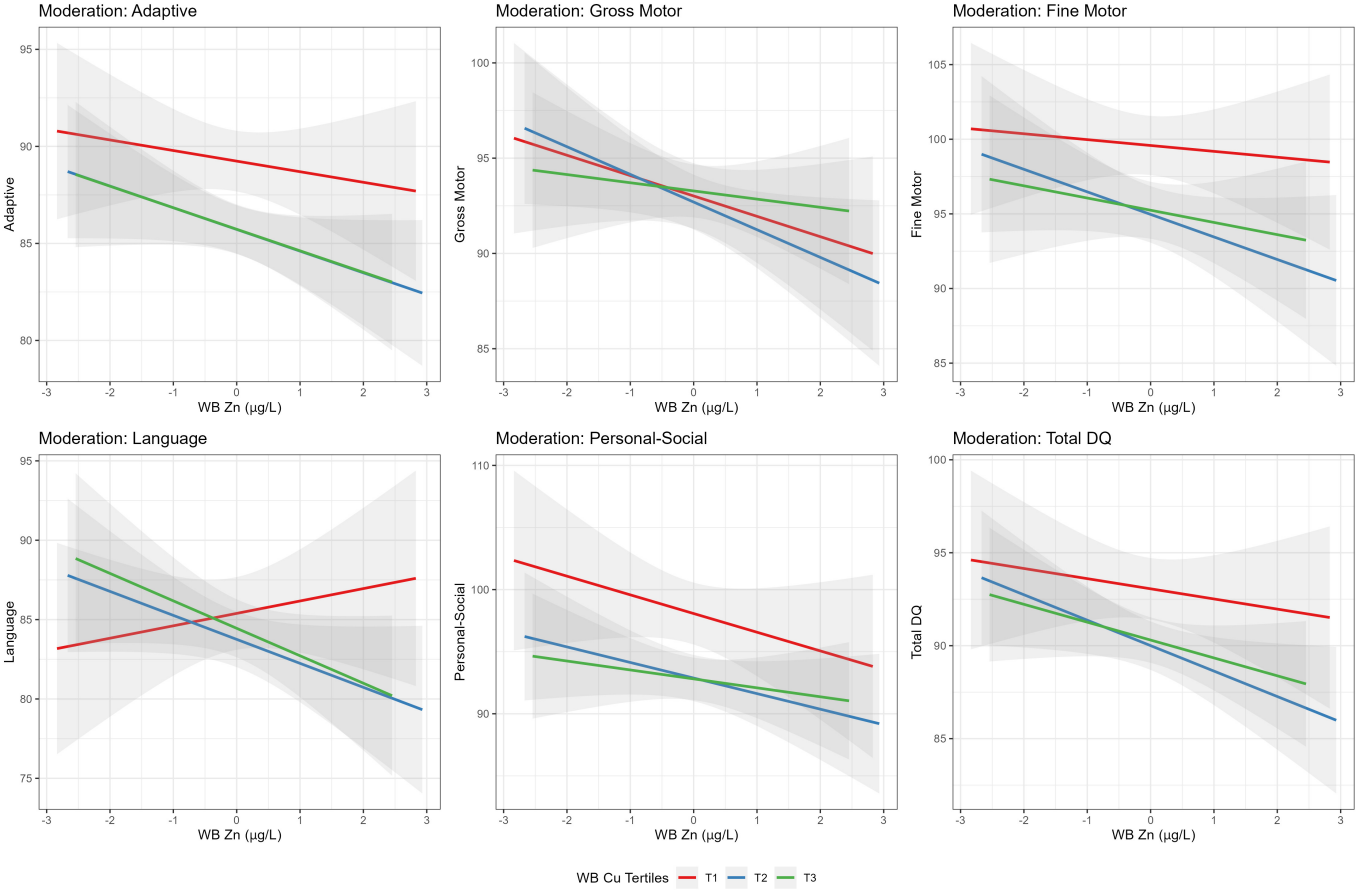
Moderation effect of whole blood copper levels on the associations between whole blood zinc and child developmental domains. Linear regression plots illustrate the associations between whole blood zinc concentrations (standardized) and developmental quotients across six domains: adaptive, gross motor, fine motor, language, personal-social, and total DQ. The associations are stratified by tertiles of whole blood copper: Low Cu (T1, red line), Medium Cu (T2, blue line), and High Cu (T3, green line). Shaded regions indicate 95% confidence intervals. All models were adjusted for child age, sex, maternal education, and gestational age. The diverging slopes suggest that the impact of zinc on development varies according to the child's copper status.

Stratified analyses (Figure 2 and Supplementary Table S4) demonstrated that the association between maternal zinc levels and child developmental scores was significantly moderated by maternal copper status. To formally quantify this modifying effect, statistical testing of the multiplicative interaction term was performed, yielding specific P_interaction_ values (Supplementary Table S4). These formal stratified models revealed that zinc exhibited a clear neuroprotective asset and was positively correlated with language development specifically under low copper conditions, whereas this beneficial effect was progressively attenuated or reversed at higher copper levels. Consistent with these findings, the stratified RCS matrices (Supplementary Figure S5) illustrated that the dose-response trajectories and specific inflection points of zinc's impact on child developmental quotients shifted dramatically across different maternal copper strata, reinforcing the necessity of adopting the integrated Cu/Zn ratio as a more reliable predictive biomarker.

### 3.2 Validation of neurotoxic effects in animal models

#### 3.2.1 Establishment of dosage-dependent animal models

To explore the biological plausibility of the associations observed in the population cohort, we established four experimental groups of SD rats modeled on different Cu/Zn ratios: a Control group (optimal ratio), a Low Cu/Zn ratio group (severe Cu deficiency), a Medium Cu/Zn ratio group (Zn-induced secondary Cu deficiency), and a High Cu/Zn ratio group (chronic Cu overload). To confirm that these dietary interventions effectively translated into systemic exposure, whole blood trace element concentrations were quantified. As the reviewer astutely noted regarding physiological intestinal compensatory mechanisms, our data revealed distinct systemic profiles. Interestingly, the maternal High Cu/Zn ratio group maintained a normal systemic Cu/Zn ratio (0.208), comparable to the Control group (0.202), demonstrating robust intestinal regulation against dietary copper overload. Conversely, the Medium Cu/Zn ratio group (designed to induce secondary imbalance via zinc overload) successfully bypassed this regulation, resulting in a profoundly elevated actual whole-blood Cu/Zn ratio in pregnant dams (0.402). This was driven by a significant systemic accumulation of copper (1555.96 ± 34.91 vs. 990.67 ± 36.06 μg/L in Controls) alongside depleted zinc levels (3869.06 ± 66.48 vs. 4911.97 ± 924.80 μg/L). Detailed blood concentrations for both dams and offspring are provided in Supplementary Table S7. This powerful systemic validation explains why the most significant neurodevelopmental and behavioral alterations were observed specifically in the Medium Cu/Zn ratio group, suggesting that offspring outcomes are a direct consequence of actual maternal systemic blood imbalance rather than mere dietary intake.

#### 3.2.2 Effects on offspring growth and cognitive function

**Figure 3:**
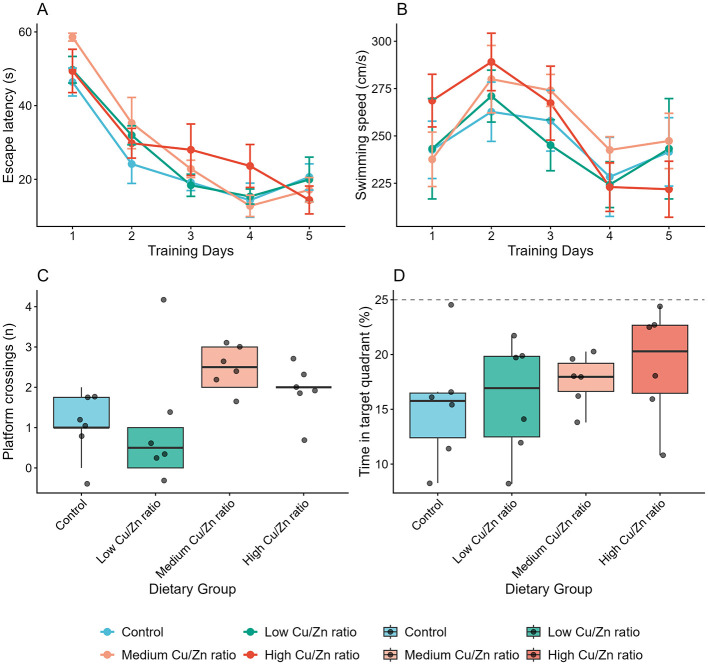
Impairment of Spatial Learning and Memory in Offspring SD Rats. **(A)** Mean escape latency during the 5-day orientation navigation trials, representing spatial learning acquisition. **(B)** Number of platform crossings in the spatial probe test on day 6, evaluating spatial memory retention. **(C)** Representative swimming tracks of each group during the probe trial. Both high and low maternal Cu/Zn ratios significantly impaired cognitive performance compared to the control group. Data are presented as mean ± SEM (*n* = 6 per group). Statistical significance was determined by repeated-measures two-way ANOVA for escape latency and one-way ANOVA followed by Tukey's *post-hoc* test for platform crossings6. *P < 0.05 vs. Control.

Longitudinal analysis (Supplementary Figure S6) revealed significant deviations in body weight gain in the imbalance groups compared to controls, emerging as early as PND 7. Cognitive assessment via the Morris Water Maze (Figure 3) indicated distinct behavioral phenotypes (33). During the 5-day hidden platform training phase, all groups exhibited progressive spatial learning, evidenced by a highly significant main effect of training days on escape latency (Repeated Measures ANOVA, *P*_time_ < 0.001). However, there was no significant main effect of the dietary Cu/Zn group (*P*_group_ = 0.557) nor a significant time-by-group interaction (*P*_interaction_ = 0.271), indicating that the overall spatial learning boundaries were comparable across all groups (Figure 3A). Furthermore, an adaptive peak in swimming speed was universally observed on Day 2 as animals adapted to the water stress (Figure 3B). In the spatial probe test conducted on Day 6, we assessed memory retention and exploratory strategies. While no significant differences were observed among the groups regarding the percentage of time spent in the target quadrant (One-way ANOVA, *P* = 0.540; Figure 3D), the number of platform crossings showed a statistically significant variation across the groups (*P* = 0.042; Figure 3C). Specifically, the Medium and High Cu/Zn ratio groups exhibited altered platform crossing behaviors, suggesting that specific thresholds of Cu/Zn imbalance may induce distinct exploratory phenotypes rather than simple memory retention deficits”

### 3.3 Proteomic profiling of the offspring brain

#### 3.3.1 Identification of the core proteomic signature

**Figure 4:**
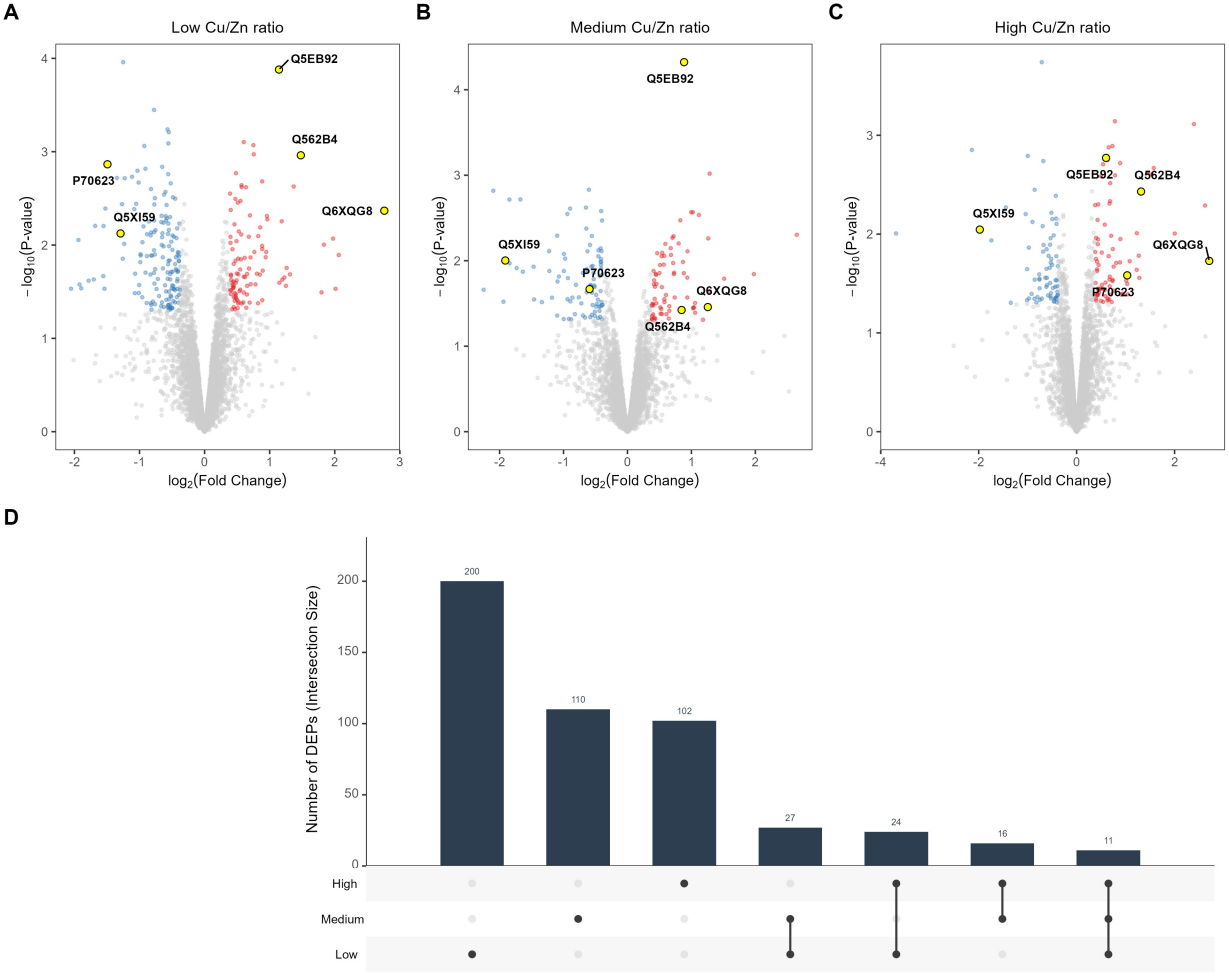
Proteomic landscapes and intersection analysis of DEPs across Cu/Zn ratios **(A–C)** Volcano plots illustrating the differentially expressed proteins (DEPs) in the Low, Medium, and High Cu/Zn ratio groups compared to the Control group (Thresholds: *P* < 0.05 and |log_2_FC| > 0.38). Red dots represent up-regulated proteins, while blue dots represent down-regulated proteins. Key neurodevelopmental markers (e.g., Q5EB92, Q5XI59) are highlighted in yellow. **(D)** UpSet plot illustrating the intersection of DEPs across the three experimental groups. A core set of 11 proteins was identified as consistently dysregulated regardless of the Cu/Zn ratio.

We performed TMT-based quantitative proteomics on brain tissues to investigate the molecular mechanisms driven by maternal Cu/Zn imbalance (34). Volcano plots (Figures 4A–C) revealed distinct proteomic landscapes in the Low, Medium, and High Cu/Zn ratio groups compared to the Control (Thresholds: *P* < 0.05 and |log_2_FC| > 0.38). Despite the divergent treatments, Intersection analysis via an UpSet plot (Figure 4D) identified a “core signature” of 11 common DEPs that were consistently dysregulated across all three imbalance groups.

#### 3.3.2 Dosage-response analysis of key neurodevelopmental markers

**Figure 5:**
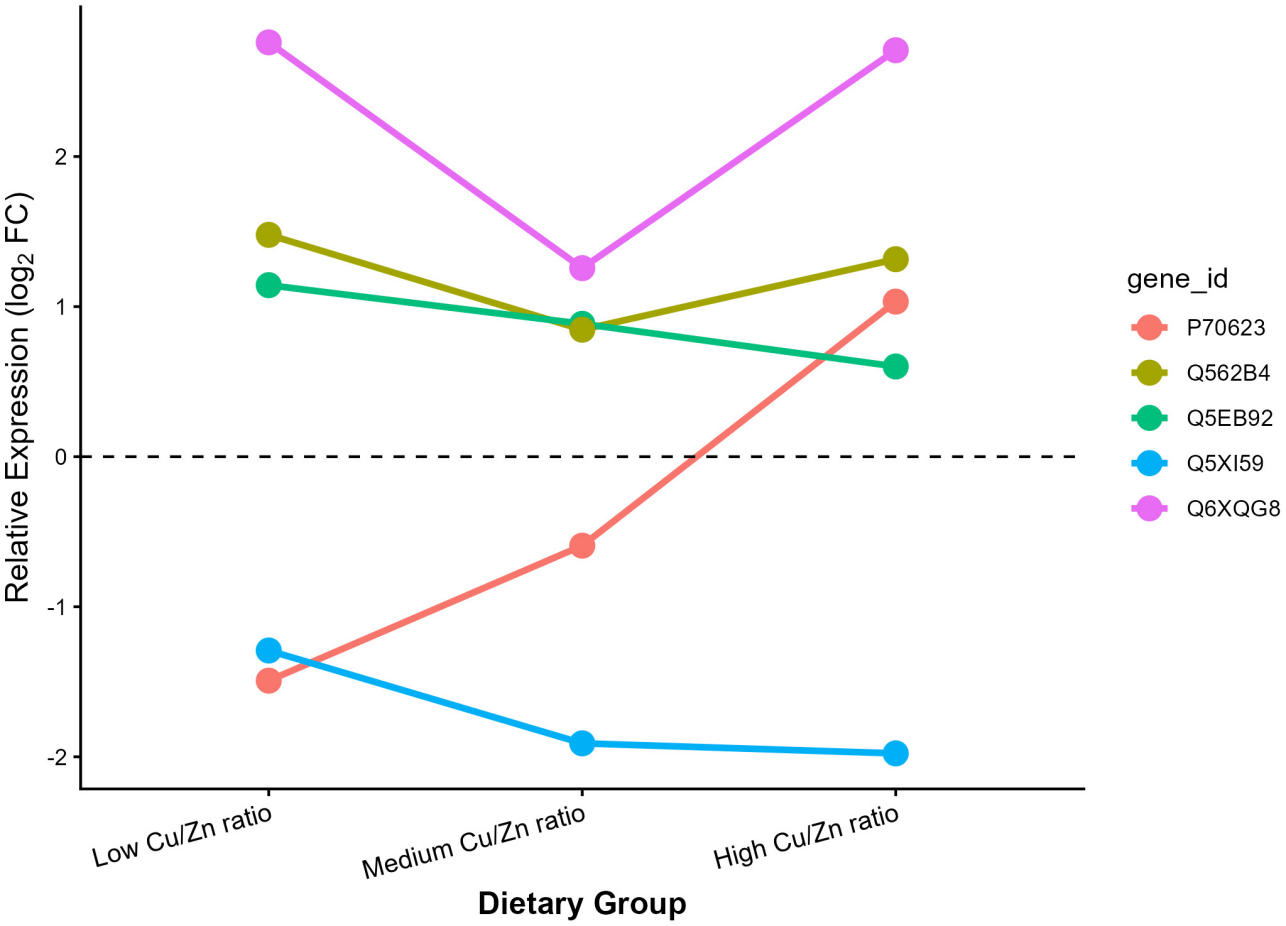
Expression trends of key neurodevelopmental proteins. Log_2_ fold change (Log_2_FC) expression profiles of five representative core proteins (SLC1A3, KCC2, MAP2, SYT1, and NDUFB8) across the Low, Medium, and High Cu/Zn ratio groups. The trend lines illustrate the diverse molecular responses of the offspring brain to maternal Cu/Zn ratio fluctuations, highlighting potential dosage-dependent effects on neuronal homeostasis.

From the 11 core DEPs, we selected five proteins essential for synaptic homeostasis and neuronal integrity to analyze expression trends (Figure 5).

##### 3.3.2.1 Inhibitory circuit maturation (KCC2/SLC12A5)

The potassium-chloride cotransporter KCC2 (Q5EB92), critical for the GABAergic excitatory-to-inhibitory switch, was upregulated in all imbalance groups. However, it exhibited a decreasing trend as the Cu/Zn ratio increased, with the highest expression levels observed in the Low Cu/Zn ratio group (log_2_FC ≈ 1.1) and the lowest in the High Cu/Zn ratio group.

##### 3.3.2.2 Synaptic vesicle function (SYT1)

The synaptic vesicle sensor Synaptotagmin-1 (Q6XQG8) displayed a V-shaped expression pattern. It was strongly upregulated in both the Low and High Cu/Zn ratio groups (peaking above log_2_FC > 2.5) but showed a marked dip in the Medium Cu/Zn ratio group (log_2_FC ≈ 1.3).

##### 3.3.2.3 Neuronal structure (MAP2)

The dendritic marker MAP2 (P70623) showed a distinct linear-like increase across the groups. It was downregulated in the Low Cu/Zn ratio group (log_2_FC ≈ −1.5), moderately downregulated in the Medium Cu/Zn ratio group (log_2_FC ≈ −0.6) and shifted to upregulation in the High Cu/Zn ratio group (log_2_FC ≈ 1.0).

##### 3.3.2.4 Excitatory & metabolic homeostasis (SLC1A3 & NDUFB8)

The glutamate transporter SLC1A3 (Q5XI59) was consistently downregulated across all groups (blue line), while the mitochondrial subunit NDUFB8 (Q562B4) remained consistently upregulated (olive line).

#### 3.3.3 Functional enrichment and biological mapping

**Figure 6:**
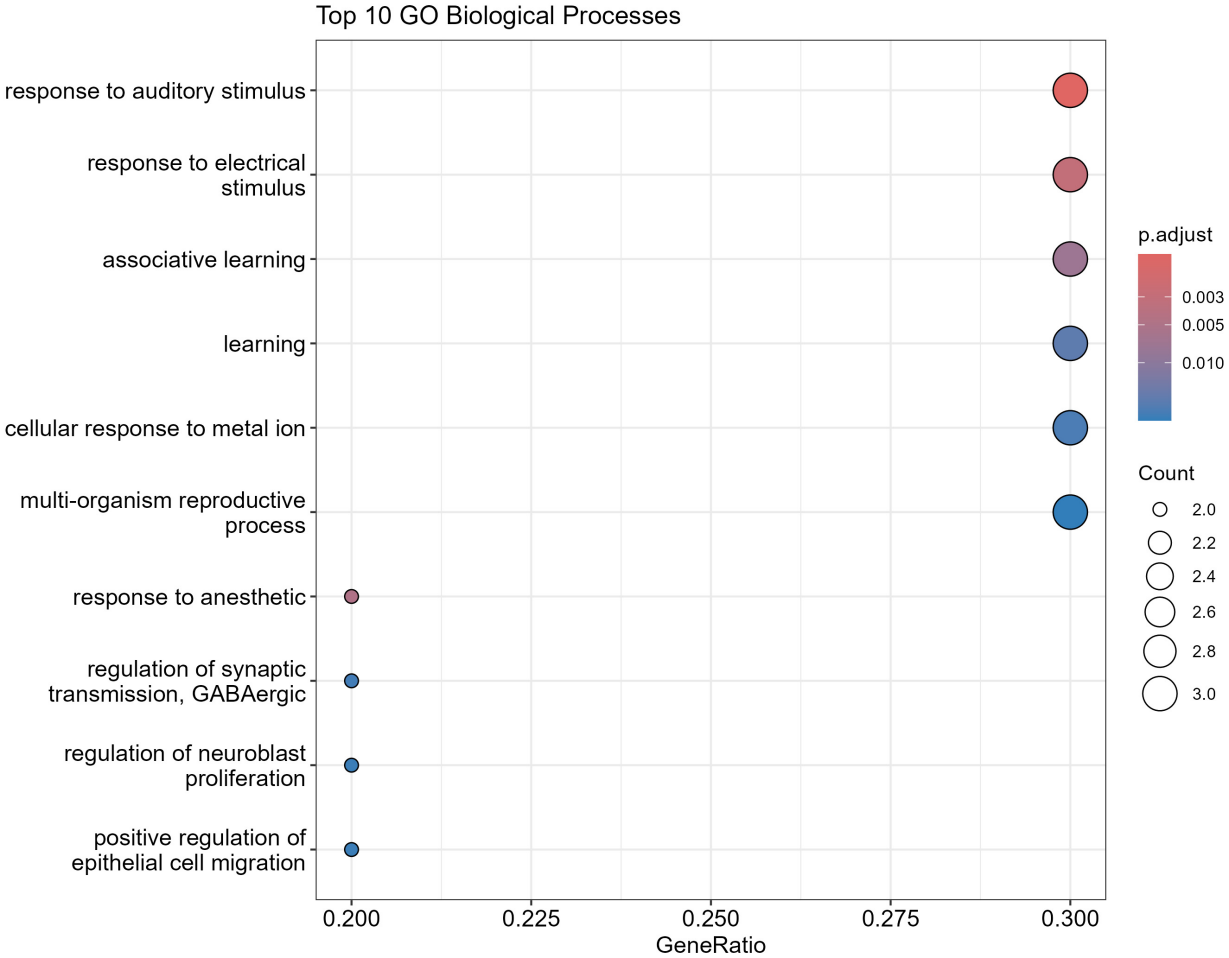
Functional Enrichment Analysis of the Common DEPs. Dot plot showing the Top 10 Gene Ontology (GO) Biological Processes enriched by the 11 common DEPs. The results highlight critical pathways including associative learning, cellular response to metal ions, and regulation of GABAergic synaptic transmission. The color intensity represents the adjusted *P*-value, and the size of the dots indicates the gene count within each term.

To integrate these molecular changes into a functional framework, GO enrichment analysis was performed on the core DEPs (Figure 6). The analysis revealed significant clustering in biological processes related to “associative learning”, “cellular response to metal ions”, and “regulation of GABAergic synaptic transmission”. To further elucidate the functional interplay and topological organization among the 11 core differentially expressed proteins (DEPs) consistently dysregulated across all treatment groups, a Protein-Protein Interaction (PPI) network was constructed using the STRING database (version 12.0) with a confidence score threshold of >0.4.

The resulting network revealed a highly interconnected and tightly clustered molecular architecture predominantly centered around synaptic integrity. Based on network topology analysis, SLC1A3 (glutamate transporter), SLC12A5 (KCC2, potassium-chloride cotransporter), SYT1 (synaptotagmin-1), and MAP2 (microtubule-associated protein 2) emerged as the predominant key hub proteins within the system, exhibiting the highest node degrees and betweenness centrality. These central hubs tightly bridge independent biological modules—specifically pairing excitatory-inhibitory neurotransmission with synaptic vesicle trafficking and dendritic structural stability. The synchronized disruption of these core hubs provides a compelling molecular rationale for the altered exploration patterns and spatial search behaviors observed in the offspring.

## 4 Discussion

In this study, we present a comprehensive analysis exploring how maternal zinc, copper, and their relative ratio during pregnancy are associated with early childhood neurodevelopment (ages 2–3 years). Our findings demonstrate that the maternal whole blood level of Cu and the Cu/Zn correlate negatively with child neurodevelopment. Zinc levels are positively associated with language development in the context of low copper. Specifically, zinc exhibited a clear neuroprotective association with developmental scores under low copper conditions; however, this beneficial effect was markedly attenuated or even reversed at higher maternal copper concentrations. The association between Zn and neurodevelopment was significantly moderated by maternal Cu levels, whereas high Cu levels appeared to attenuate the neuroprotective potential of Zn. Animal experiments mirrored these findings, showing that specific Cu/Zn imbalances (particularly the Medium Cu/Zn ratio) were associated with behavioral alterations indicative of altered exploratory pattern in offspring. Proteomic analyses suggest these imbalances disrupt synaptic homeostasis and neuronal structural integrity.

Compared with previous population-based studies, our findings are both consistent with and extend existing evidence, such as the Spanish INMA cohort have reported that higher maternal copper exposure is associated with delayed cognitive and motor development in early childhood (37), which is broadly in line with our observation that elevated maternal Cu levels were associated with less favorable neurodevelopmental outcomes. In addition, the Cu/Zn ratio has increasingly been proposed as a potential biomarker of systemic oxidative stress and inflammation (38). This may provide a plausible explanation for the observed association between higher Cu/Zn ratios and poorer developmental performance in our cohort.

However, regarding the independent effect of zinc, several cohorts in Western countries and other regions of China (such as eastern coastal areas) have generally described prenatal zinc exposure as a protective factor for neurodevelopment (39, 40). In contrast, our findings suggest a more complex pattern characterized by non-linear and copper-dependent associations. Specifically, zinc appeared to be beneficial under relatively low copper conditions, whereas this association became attenuated or even reversed at higher copper levels.

Several factors may contribute to these differences. One possible explanation is the substantial variation in baseline trace element exposure levels across populations. Our cohort was conducted in Guangxi, China, a region characterized by karst geology and relatively high background levels of non-ferrous metals. As a result, the maternal trace element profile in our cohort exhibits a significant upward baseline shift compared to populations from non-mining regions or Western countries (23, 24). Specifically, the median (50th percentile) whole blood concentrations of copper and zinc in our cohort reached 1418.19 μg/L and 5123.99 μg/L, respectively, with the highest zinc tertile exceeding 5563.6 μg/L. To contextualize this geochemical enrichment, these metrics are notably higher than those documented in standard low-exposure populations; for instance, the median maternal whole blood copper and zinc levels reported in a cohort of non-occupationally exposed pregnant women from Hangzhou, China, were approximately 451 μg/L and 1,782 μg/L, respectively (41). Consistent with our RCS analyses, the relationship between trace element exposure and neurodevelopment may follow a non-linear pattern with potential threshold effects. In populations with relatively low zinc exposure, individuals may predominantly fall within the ascending segment of the dose–response curve, where zinc exerts beneficial effects. In contrast, the elevated baseline exposure in our cohort allowed us to capture a much wider exposure spectrum. This unique epidemiological window enabled us to observe a proportion of individuals who approach or exceed the optimal physiological range, successfully unmasking the non-linear, downward trajectories where excessive copper or Cu/Zn imbalance plateaus or even reverses the beneficial associations. Consequently, this distinct high-exposure profile not only explains the divergence of our findings from traditional cohorts but also fundamentally strengthens the external validity of our results, extending their translational relevance to other highly mineralized, karst, or mining-intensive regions globally.

In addition to environmental exposure differences, variation in dietary patterns and genetic background may further contribute to the observed heterogeneity. For instance, regional dietary habits—such as the consumption of locally grown foods enriched in trace elements— may influence maternal zinc and copper intake. Moreover, genetic polymorphisms involved in metal metabolism, including those related to metallothionein pathways, may affect the absorption, distribution, and regulation of these trace elements. Such inter-individual differences may partly account for the variability in Cu/Zn balance and its association with neurodevelopmental outcomes observed in our study (42).

Behavioral assessments using the Morris water maze indicated that baseline spatial learning and memory retention were generally preserved across all groups. Notably, during the spatial probe trial, the Medium Cu/Zn ratio group unexpectedly exhibited significantly increased platform crossings. While higher platform crossings traditionally indicate robust memory retention, it is highly plausible that the standard Morris water maze paradigm may not be the most appropriate or sensitive methodology to capture the specific, nuanced neurocognitive alterations reported in our human cohort. However, although the *in vivo* behavioral experiment did not overtly unmask the anticipated phenotypic deficits, our subsequent proteomic profiling data points in a direction consistent with the human study's findings. The profound dysregulation of critical synaptic markers (such as SLC1A3, KCC2, and SYT1) under Cu/Zn imbalance demonstrates severe underlying molecular disturbances. This molecular evidence provides crucial biological plausibility for our epidemiological observations, suggesting that these synaptic alterations might require alternative, more specific behavioral testing paradigms to be fully translated at the phenotypic level. Taken together, although the behavioral outcomes did not fully recapitulate the neurodevelopmental patterns observed in the human cohort, the consistent synaptic protein alterations across Cu/Zn imbalance groups provide molecular evidence that supports the biological plausibility of our epidemiological findings.

At the molecular level, proteomic analysis identified 11 differentially expressed proteins (DEPs) associated with elevated Cu/Zn ratios. Functional enrichment analysis indicated that these proteins were involved in pathways related to associative learning and the regulation of GABAergic synaptic transmission, highlighting potential disturbances in synaptic signaling processes.

Several proteins involved in synaptic regulation were particularly affected. For instance, KCC2 plays a key role in the developmental transition of GABAergic signaling from excitatory to inhibitory (43), whereas SLC1A3 (EAAT1) contributes to the clearance of glutamate from the synaptic cleft (44). Altered expression of these proteins under high Cu/Zn conditions may influence the balance between excitatory and inhibitory neurotransmission. In addition, changes in MAP2 and SYT1 expression may reflect alterations in dendritic structural organization and synaptic vesicle release processes (45). We also observed modulation of NDUFB8, a subunit of mitochondrial complex I, which may indicate involvement of mitochondrial function and oxidative stress pathways (46).

Furthermore, regarding the underlying molecular mechanisms, it is highly plausible that Metallothionein-3 (MT-3) plays a pivotal role in mediating the neurodevelopmental effects of trace element imbalance. As astutely hypothesized, elevated systemic copper or a high Cu/Zn ratio might induce MT-3 overexpression, which is known to inhibit neurite outgrowth and could partly explain the altered spatial exploration phenotypes observed in our animal model. In our current quantitative proteomics dataset, MT-3 (UniProt ID: P37361) was indeed successfully identified; however, its relative abundance did not exhibit a statistically significant difference between the High Cu/Zn and Control groups (*P* = 0.730). This absence of statistical significance likely stems from the inherent technical limitations of conventional bottom-up proteomics platforms. MT-3 is a low-molecular-weight (~6.8 kDa) and extremely cysteine-rich protein, rendering it notoriously difficult to efficiently digest, ionize, and precisely quantify using standard LC-MS/MS pipelines. Therefore, while our non-targeted omics approach did not capture a definitive up-regulation, the MT-3 pathway remains a strong mechanistic candidate. Future studies employing targeted, low-throughput validation techniques, such as Western blotting or targeted mass spectrometry, are warranted to definitively test the MT-3 hypothesis in the context of prenatal Cu/Zn imbalance.

Taken together, these findings suggest that an elevated Cu/Zn ratio may influence neurodevelopment through multiple biological pathways, including oxidative stress responses, synaptic signaling regulation, and neuronal structural stability. These mechanistic observations provide supportive biological context for the epidemiological associations observed in our human cohort.

The present study possesses several notable strengths. First, the population-based prospective cohort design allows for a robust temporal evaluation between prenatal trace element exposures and subsequent neurodevelopmental outcomes in early childhood. This longitudinal approach significantly minimizes recall bias and strengthens the basis for causal inference compared to cross-sectional study designs. Second, to the best of our knowledge, this is the first study to provide comprehensive epidemiological evidence highlighting the interactive effect—rather than merely the independent effects—of maternal copper and zinc on neurodevelopment. By utilizing non-linear dose-response models and stratified analyses, we characterized the complex moderation effect of copper on zinc's neuroprotective potential, thereby filling a critical knowledge gap in maternal-fetal nutrition. Finally, a major methodological strength of this study is the integration of epidemiological observations with *in vivo* mechanistic exploration. By bridging the human cohort data with a Cu/Zn-imbalanced rodent model and advanced proteomic analysis, we provide biological plausibility for our observational findings. The identification of key synaptic targets offers novel molecular insights and a foundation for future experimental research on how Cu/Zn interactions may influence neurodevelopmental trajectories.

Despite the aforementioned strengths, several limitations of this study should be acknowledged. First, although our prospective design minimized temporal biases, the relatively moderate sample size of our cohort (*n* = 725) may have limited our statistical power, particularly when detecting subtle effects within the stratified analyses or evaluating higher-order interactions. Larger, multicenter population-based cohorts are warranted to validate our non-linear findings and allow for more granular subgroup evaluations. Second, a primary limitation of this study is that the assessment of zinc and copper was restricted to internal exposure levels, with a corresponding lack of external exposure data. Although food frequency questionnaires were collected, the absence of 24-h dietary recall data precluded the quantification of specific dietary intakes for zinc and copper. Finally, while our animal model and proteomic profiling successfully provided essential biological plausibility for the epidemiological observations, the mechanistic exploration remains preliminary. Although we identified downstream dysregulations in synaptic homeostasis—such as the disruption of KCC2 and SLC1A3—the exact upstream signaling cascades and the precise spatiotemporal localization of these molecular events in the developing brain require further delineation. Future experimental studies incorporating targeted gene manipulations, *in vivo* electrophysiology, or metabolomics are needed to comprehensively map the neurotoxic pathways driven by Cu/Zn imbalances.

Furthermore, while this study meticulously focused on the physiological antagonism of the Cu/Zn axis, we acknowledge that the central nervous system is exposed to a broader mixture of essential and toxic microminerals. Given the high background levels of non-ferrous metals in the karst and mining regions of our study population, elements such as manganese (Mn) are also of critical developmental importance. It is highly plausible that Mn levels might interact with the Cu/Zn homeostatic network. Although investigating the complete multi-metal interactome is beyond the scope of our matched *in vivo* Cu/Zn animal model, delineating the complex interactions between the Cu/Zn ratio and other region-specific microminerals (particularly Mn) remains a highly valuable direction for our future cohort investigations.

## 5 Conclusion

In conclusion, our findings suggest that the maternal mid-pregnancy Cu/Zn ratio is non-linearly associated with offspring neurodevelopmental outcomes at 2–3 years of age, with potential biological links to synaptic protein alterations as observed in our animal model. Moving beyond generalized recommendations of trace element “balance,” we offer a preliminary conceptual framework for personalized prenatal nutritional assessment. This framework highlights the value of concurrent dual-index monitoring (absolute concentrations and Cu/Zn ratio), calibrating nutritional profiles against regional geochemical baselines, and tailoring prenatal zinc supplementation to individual maternal copper strata to prevent antagonistic effects. However, given the study's observational and exploratory design, future large-scale prospective trials are necessary to validate these preliminary concepts before clinical implementation.

## Data Availability

The mass spectrometry proteomics data have been deposited to the ProteomeXchange Consortium via the iProX partner repository with the dataset identifier PXD080627 and can be accessed at https://www.iprox.cn//page/project.html?id=IPX0018107000. The raw data supporting the conclusions of this article will be made available by the authors without undue reservation to any qualified researcher. The human cohort data are not publicly available due to ethical restrictions and privacy protection requirements for participants' personal health information, but may be obtained from the corresponding author upon reasonable request and with appropriate institutional ethical approval.
