# Supplementary material for: Maternal copper levels moderate the neuroprotective effect of zinc on early childhood development: evidence from a birth cohort and proteomic profiling
Source: Front Nutr. 2026 Jul 13;13:1868249. doi: 10.3389/fnut.2026.1868249 (PMC13403798; doi:10.3389/fnut.2026.1868249)
Supplement: Supplementary file 1 [file Table_1.docx]

**S1. Flowchart of study participant selection.**

**
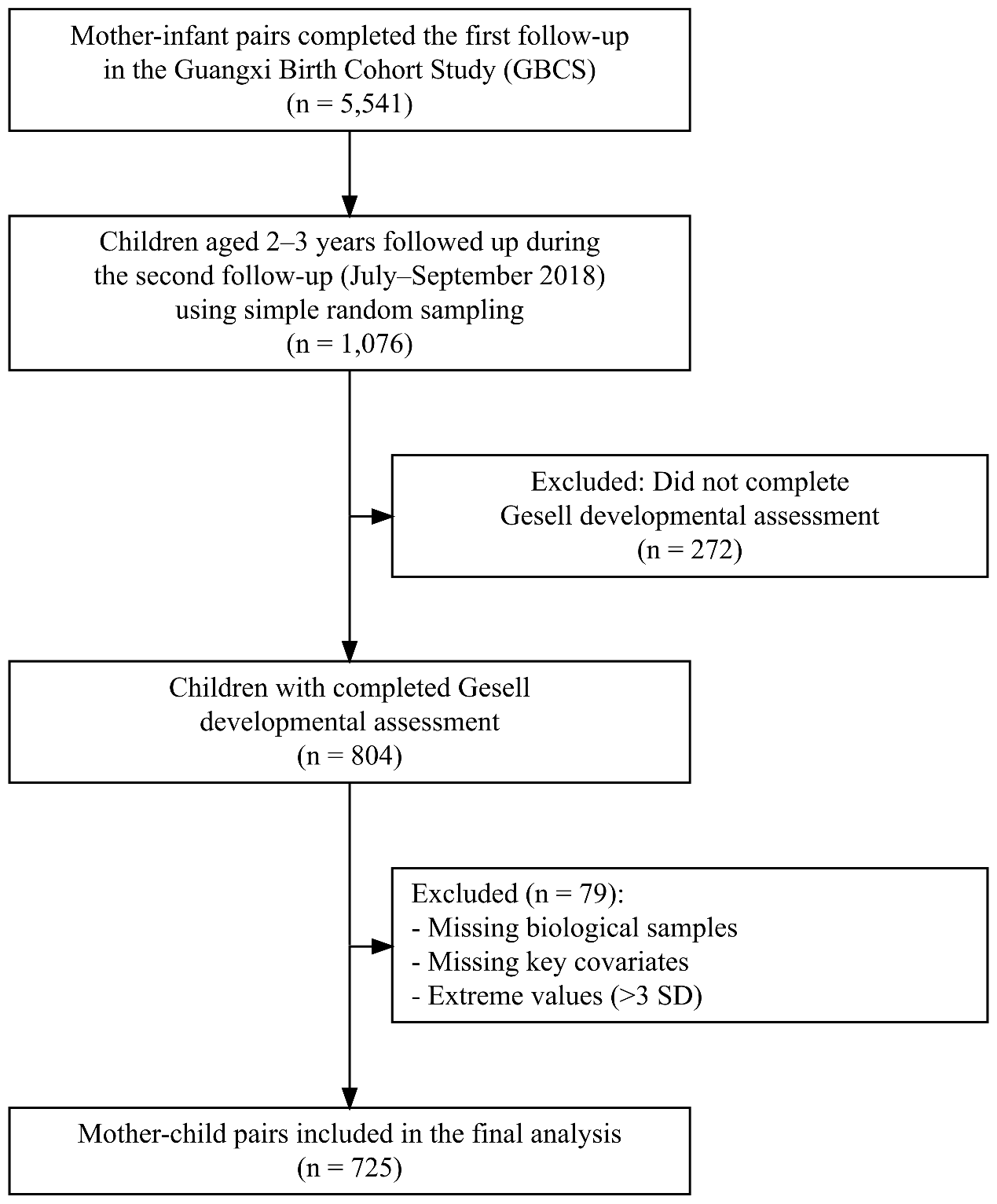
**

*Figure S1:* *This study was embedded within the Guangxi Birth Cohort Study (GBCS). From the 5,541 mother–infant pairs who completed the first follow-up, 1,076 children aged 2–3 years were followed up during the second follow-up (July–September 2018) using simple random sampling. Among them, 804 children completed the Gesell developmental assessment. After excluding 79 participants due to missing maternal serum trace element data, missing key covariates, or extreme metal concentrations (>3 SD), a total of 725 mother–child pairs were included in the final analysis.*

**S2. Demographic and developmental characteristics of participants stratified by maternal whole blood zinc tertiles (*n* = 725)**

| **Variable** | **Low Tertile**  **(<4732.4)** | **Mid Tertile**  **(4732.4–5563.6)** | **High Tertile**  **(>5563.6)** | **P-value** |
| --- | --- | --- | --- | --- |
| Maternal characteristics |  |  |  |  |
| Age (years) | 28.2 ± 4.6 | 28.5 ± 4.9 | 29.0 ± 4.3 | 0.223 |
| Pre-pregnancy BMI (kg/m²) | 20.4 ± 3.2 | 20.6 ± 3.3 | 20.7 ± 2.9 | 0.504 |
| Educational level, n (%) |  |  |  | 0.858 |
| Junior high school or below | 68 (28.1) | 71 (29.5) | 70 (28.9) |  |
| Senior high school | 70 (28.9) | 75 (31.1) | 66 (27.3) |  |
| College or above | 104 (43.0) | 95 (39.4) | 106 (43.8) |  |
| Annual household income, n (%) |  |  |  | 0.100 |
| Low | 147 (60.7) | 151 (62.7) | 125 (51.7) |  |
| Medium | 73 (30.2) | 72 (29.9) | 96 (39.7) |  |
| High | 22 (9.1) | 18 (7.5) | 21 (8.7) |  |
| Alcohol use before pregnancy, n (%) |  |  |  | 0.231 |
| Yes | 59 (24.4) | 60 (24.9) | 74 (30.6) |  |
| No | 183 (75.6) | 181 (75.1) | 168 (69.4) |  |
| Passive smoking during pregnancy, n (%) |  |  |  | 0.109 |
| Yes | 24 (9.9) | 39 (16.2) | 29 (12.0) |  |
| No | 218 (90.1) | 202 (83.8) | 213 (88.0) |  |
| Gestational week at sampling | 19.6 ± 6.4 | 18.7 ± 6.8 | 19.2 ± 6.9 | 0.286 |
| Child characteristics |  |  |  |  |
| Sex (male), n (%) | 149 (61.6) | 143 (59.3) | 150 (62.0) | 0.814 |
| Gestational age at delivery (weeks) | 38.7 ± 1.3 | 38.6 ± 1.4 | 38.5 ± 1.5 | 0.527 |
| Age (years) | 2.6 ± 0.1 | 2.6 ± 0.1 | 2.6 ± 0.2 | 0.798 |
| Birth head circumference (cm) | 32.9 ± 1.5 | 32.8 ± 1.4 | 32.5 ± 1.7 | 0.017 |
| Developmental Quotient (DQ) |  |  |  |  |
| Adaptive behavior | 89.3 ± 12.3 | 85.7 ± 9.8 | 85.8 ± 10.1 | <0.001 |
| Gross motor | 93.1 ± 13.6 | 92.7 ± 11.4 | 93.2 ± 10.9 | 0.905 |
| Fine motor | 99.6 ± 15.6 | 94.9 ± 14.9 | 95.3 ± 15.1 | <0.001 |
| Language | 85.4 ± 18.1 | 83.7 ± 13.8 | 84.5 ± 14.5 | 0.492 |
| Personal-social behavior | 98.1 ± 19.7 | 92.9 ± 14.8 | 92.8 ± 13.4 | <0.001 |
| Total DQ | 93.1 ± 13.0 | 90.0 ± 10.4 | 90.3 ± 9.7 | 0.004 |

*Notes: 1. Values are presented as Mean ± SD for continuous variables and n (%) for categorical variables. 2. P-values for continuous variables were determined by one-way ANOVA, and for categorical variables by Pearson’s Chi-square test. 3. Maternal whole blood zinc concentrations were divided into tertiles: Low (<4732.4 μg/L), Mid (4732.4–5563.6 μg/L), and High (>5563.6 μg/L).*

**S3. Distribution of Whole Blood Metal Concentrations and Copper-to-Zinc Ratio among Study Participants (n = 725)**

| **Analyte** | **Detection Frequency**  **(%)** | **Limit of Detection**  **(μg/L)** | **P25​** | **P50​**  **(Median)** | **P75​** |
| --- | --- | --- | --- | --- | --- |
| Copper (Cu) | 100 | 0.055 | 4487.49 | 5123.99 | 5874.48 |
| Zinc (Zn) | 100 | 0.507 | 1252.96 | 1418.19 | 1548.58 |
| Cu/Zn Ratio | — | — | 3.14 | 3.68 | 4.46 |

*Note: Units are μg/L for metal concentrations; the Cu-to-Zn ratio is unitless. P25, P50, and P75 represent the 25th, 50th (median), and 75th percentiles, respectively. “—” indicates not applicable or not available. The Cu/Zn ratio was calculated by dividing the whole blood copper concentration by the whole blood zinc concentration for each participant.*

**S4. Associations between maternal whole-blood zinc tertiles and offspring neurodevelopmental outcomes, stratified by maternal whole-blood copper baseline, with formal interaction tests.**

| **Outcome_Domain** | **Maternal_Cu_Strata** | **Tertile_1_Ref** | **Tertile_2_OR_95CI** | **Tertile_3_OR_95CI** | **P_interaction** |
| --- | --- | --- | --- | --- | --- |
| Adaptive | Low Copper Baseline (<=Median) | 1.00 (Ref) | 0.95 (0.51, 1.76) | 0.96 (0.51, 1.80) | 0.933 |
| Adaptive | High Copper Baseline (>Median) | 1.00 (Ref) | 1.06 (0.58, 1.92) | 0.97 (0.54, 1.76) | - |
| Gross Motor | Low Copper Baseline (<=Median) | 1.00 (Ref) | 0.90 (0.50, 1.64) | 1.54 (0.86, 2.76) | 0.36 |
| Gross Motor | High Copper Baseline (>Median) | 1.00 (Ref) | 1.10 (0.61, 2.00) | 1.04 (0.57, 1.91) | - |
| Fine Motor | Low Copper Baseline (<=Median) | 1.00 (Ref) | 1.66 (0.91, 3.01) | 1.24 (0.66, 2.34) | 0.571 |
| Fine Motor | High Copper Baseline (>Median) | 1.00 (Ref) | 1.08 (0.59, 1.98) | 1.31 (0.73, 2.38) | - |
| Language | Low Copper Baseline (<=Median) | 1.00 (Ref) | 0.92 (0.51, 1.66) | 0.75 (0.40, 1.41) | 0.537 |
| Language | High Copper Baseline (>Median) | 1.00 (Ref) | 0.85 (0.45, 1.58) | 1.28 (0.70, 2.34) | - |
| Personal-Social | Low Copper Baseline (<=Median) | 1.00 (Ref) | 1.27 (0.69, 2.34) | 1.52 (0.81, 2.83) | 0.597 |
| Personal-Social | High Copper Baseline (>Median) | 1.00 (Ref) | 0.95 (0.50, 1.79) | 1.42 (0.78, 2.60) | - |
| Total DQ | Low Copper Baseline (<=Median) | 1.00 (Ref) | 1.17 (0.63, 2.17) | 1.18 (0.63, 2.22) | 0.132 |
| Total DQ | High Copper Baseline (>Median) | 1.00 (Ref) | 0.58 (0.30, 1.11) | 0.99 (0.54, 1.82) | - |

*Note: OR, odds ratio; CI, confidence interval; Ref, reference group.*

*Stratification was performed based on the median concentration of maternal whole-blood copper (Low Copper Baseline: ≤ median; High Copper Baseline: > median).*

*The P for interaction (P_interaction_) was derived from the multiplicative interaction term (Whole-blood Zinc × Whole-blood Copper) included in the generalized linear models to formally quantify the modifying effect of copper.*

*All stratified models were adjusted for child age, child sex, maternal age, pre-pregnancy BMI, maternal education, household income, birth head circumference, and gestational age at delivery.*

**S5. Stratified dose-response relationships between whole blood zinc and developmental quotients across whole blood copper tertiles.**


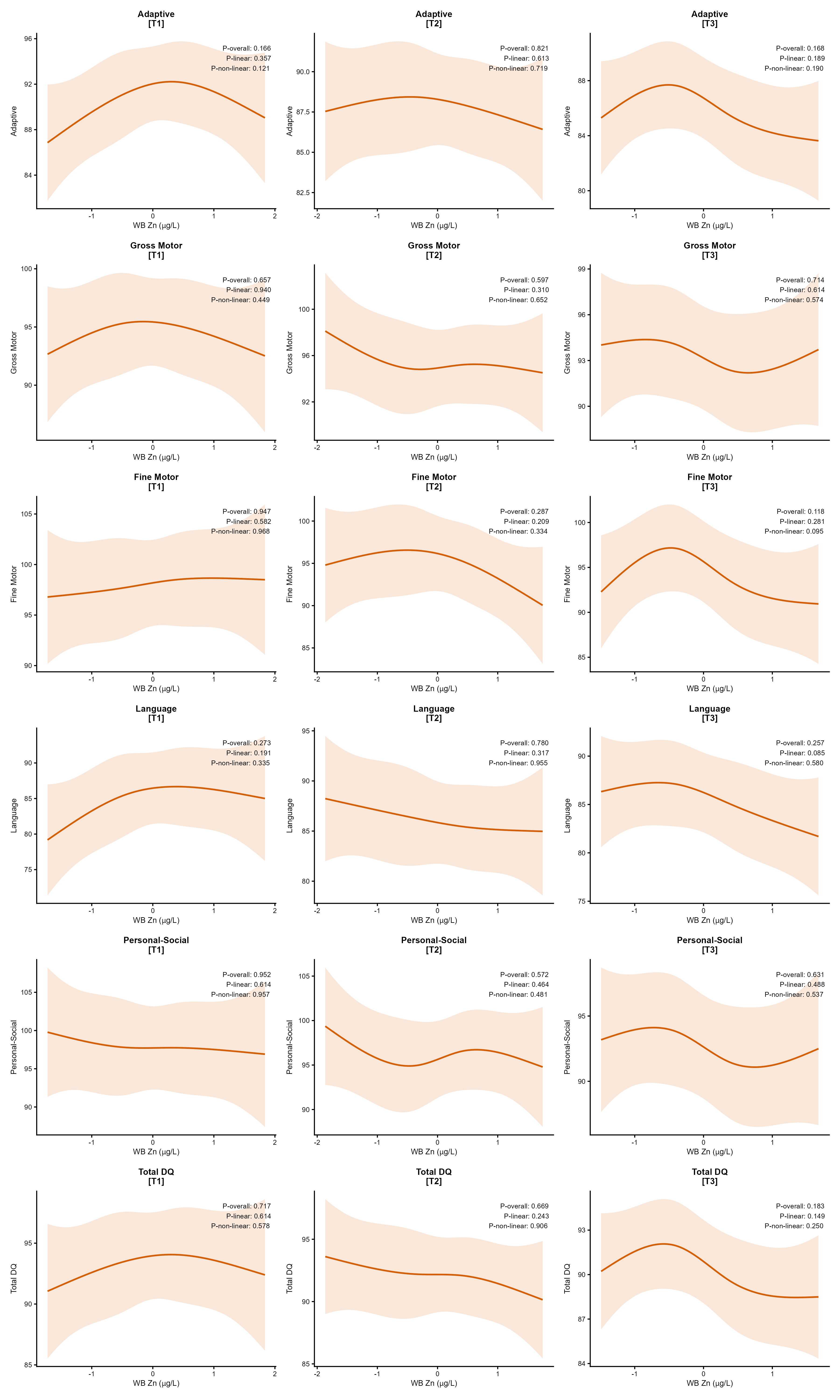


*Figure S5: A matrix of restricted cubic spline (RCS) plots illustrating the associations between whole blood zinc concentrations (standardized) and six developmental domains (rows: Adaptive, Gross Motor, Fine Motor, Language, Personal-Social, and Total DQ), stratified by tertiles of whole blood copper (columns: Low Cu [T1], Medium Cu [T2], and High Cu [T3]). Solid lines represent the adjusted predicted developmental scores, and shaded areas indicate 95% confidence intervals. Models were adjusted for child age, sex, maternal education, and gestational age. Three P-values are reported for each subgroup: P-overall (overall association), P-linear (linear trend), and P-non-linear (deviation from linearity). The knots were placed at the 5th, 35th, 65th, and 95th percentiles.*

**S6. Longitudinal Growth Trajectories of Offspring Body Weight from Postnatal Day**


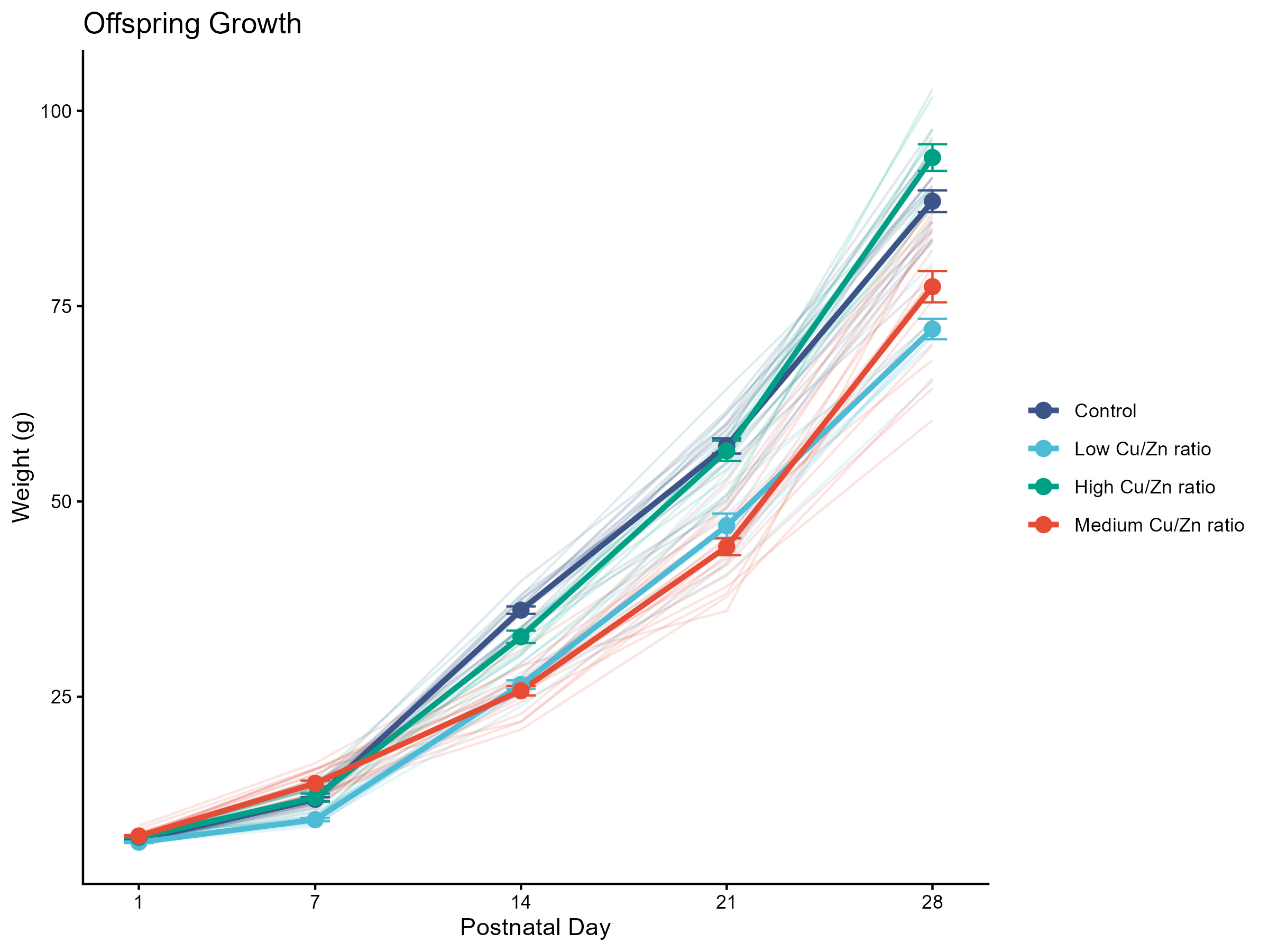


*Figure S6: Individual and group-level body weight changes of offspring rats across the four experimental groups: Control, Low Cu/Zn ratio, Medium Cu/Zn ratio, and High Cu/Zn ratio. (A–D) The "spaghetti plots" represent the longitudinal growth of each individual pup (light-colored lines) overlaid with the group mean and standard deviation (thick dark line with error bars). Weight was measured weekly on postnatal days (PND) 1, 7, 14, 21, and 28. Significant differences in growth velocity were observed among groups (refer to Table 1 for detailed statistical comparisons). n = 9–15 per group.*

**S7. Whole blood concentrations of zinc and copper in pregnant dams and offspring rats across different dietary intervention groups.**

| **Group** | **Pregnant Dams (N=2)** |  | **Offspring (PND30) (N=5)** |  |
| --- | --- | --- | --- | --- |
|  | **Zinc (μg/L)** | **Copper (μg/L)** | **Zinc (μg/L)** | **Copper (μg/L)** |
| **Control group** | 4911.97 ± 924.80 | 990.67 ± 36.06 | 4241.45 ± 241.38 | 871.81 ± 52.44 |
| **Low Cu/Zn ratio group** | 4254.61 ± 629.25 | 960.49 ± 274.47 | 3820.39 ± 585.44 | 825.05 ± 99.88 |
| **Medium Cu/Zn ratio** | 3869.06 ± 66.48 | 1555.96 ± 34.91 | 4064.78 ± 536.24 | 862.05 ± 49.76 |
| **High Cu/Zn ratio group** | 4532.60 ± 766.77 | 945.02 ± 36.44 | 3603.91 ± 747.61 | 811.05 ± 81.19 |

*Note: Data are expressed as mean ± standard deviation (SD). PND30, postnatal day 30. Whole blood trace element concentrations were determined by inductively coupled plasma mass spectrometry (ICP-MS).*
